# Consistency between education reported in health survey and recorded in death certificate

**DOI:** 10.1186/1471-2458-7-294

**Published:** 2007-10-18

**Authors:** Young-Ho Khang, Hye Ryun Kim, John W Lynch

**Affiliations:** 1 Department of Preventive Medicine, University of Ulsan College of Medicine, Seoul, Korea; 2 Korea Institute for Health and Social Affairs, Seoul, Korea; 3 Department of Epidemiology, Biostatistics and Occupational Health, McGill University, Montreal, Canada

## Abstract

**Background:**

Education level is one indicator of socioeconomic position which, in several countries including South Korea, is provided though death certificate data. Its validity determines the usefulness of death certificate data for exploring the association between socioeconomic position and mortality. This study was to compare education recorded on the death certificate with that reported before death in a nationally representative cohort of participants in the National Health and Nutrition Examination Survey (NHANES).

**Methods:**

The 1998/2001 NHANES data contained unique 13-digit personal identification numbers that were individually linked to death certificate data from the Korean National Statistical Office. Duration of mortality follow-up was 7.1 years. The data from 513 deaths were used to determine sensitivity and specificity of education in death certificate and estimate agreement rates of education level between NHANES data and death certificate data. Odds ratios for agreement in education were also estimated. Covariates considered in the analyses were gender, age, duration between NHANES and death, and cause of death.

**Results:**

The proportion of deaths without recorded education in death certificate was very low (0.2%). A total of 29.4% discordant pairs were found. Sensitivity and specificity for college or higher education were 0.84 (95% confidence interval 0.71–0.97) and 0.99 (0.98–1.00). However, sensitivity was poor for middle school education. The overall agreement rate was 70.7% (66.8%–74.6%) when education was categorized into five groups and increased up to 88.9% (86.2%–91.6%) when three education categories were used. The magnitude of validity and reliability for education did not generally vary with age, duration between health survey and death, and cause of death. However, a significantly smaller likelihood of agreement was found for middle and elementary school education after adjusting for covariates.

**Conclusion:**

Low percentage of missing information on education in South Korean death certificate data could provide a great potential to monitor mortality inequalities. A more collapsed categorization in education would be recommended when a more definitive conclusion on educational mortality inequality is required.

## Background

Education level is one indicator of socioeconomic position which, in several countries including South Korea, is provided though death certificate data. Its validity determines the usefulness of death certificate data for exploring the association between socioeconomic position and mortality [1]. Although education is likely to be more accurately reported than measures such as income, some evidence from Western studies suggests inconsistencies can occur between education reported in health surveys and that subsequently recorded on death certificates [2-4]. In a study by Lerchen and colleagues, a considerable gap was found between education reported in health interview data and education reported in subsequent follow-up interviews from surviving spouses after death [5]. Contradictory results have also been shown in other studies, with some demonstrating a tendency for education to be reported at a higher level on death certificates than at baseline health survey, [2,3] while other studies showed this was not true [1].

In addition, the reliability of reported education between interview survey and death certificate data may vary with decedent's gender, age, and educational level [3]. Education can be reported less accurately for female decedents since they are less likely than males to have surviving spouses and are more likely to die at an older age [3]. The overstatement of education was more common among those decedents who, according to health survey data, had a lower education level [3]. However, in a later study, Rosamond and colleagues reported that death certificate education level had substantial validity and that misclassification was similar in both genders [1]. Reports have shown that overstatement bias was more pronounced in older than younger decedents [2]. Apart from these studies, little has been published on the validity and reliability of education between health survey data and death certificate data.

Education is known as a socioeconomic position indicator that is less likely to change over time than occupation and income [4]. As a result, misclassification of education may not vary with the length of time between acquisition of health survey and death certificate data. However, no studies have proven this. In addition, little is known about the misclassification of education by cause of death.

Recently, several studies have been conducted to examine educational inequalities in mortality with use of census and death certificate data in South Korea [6-8]. For example, in prior studies we presented age-, gender-, and cause-specific mortality inequalities by education [6] and examined trends in mortality inequalities by education [7]. However, the possibility of numerator-denominator bias [9] could not be excluded in these studies since unlinked data were used [6,7]. If education levels of the census were more inflated than those of death certificate data, exaggerated educational mortality differentials could be made. Son claimed that the reliability of education level between survey and death certificate data can be poor [10]. A prior Korean study examined the reliability of reported education level between survey and death certificate data [11]. However, due to the small number of deaths, a more detailed analysis by covariates such as cause of death and duration between health survey and death was not possible and the validity (sensitivity and specificity) of each educational category was not examined. Information on gender, age, and cause-specific level of validity is useful to assess any bias in the gender, age, and cause-specific magnitude of socioeconomic mortality inequalities measured with census and death certificated data. Therefore, the purpose of this study was to compare education level recorded on death certificates with that reported before death in a nationally representative cohort of participants in the National Health and Nutrition Examination Survey (NHANES) of South Korea. Specifically, we examined the difference in the magnitude of validity and reliability for educational attainment according to gender, age group, duration between health survey and death, educational category, and cause of death.

## Methods

The Institutional Review Board of the Asan Medical Center, Seoul, approved this study. The health survey data used in this study were the 1998 and 2001 NHANES conducted by the Korea Institute for Health and Social Affairs. Information was collected from a stratified multistage probability sample of South Korean households representing the civilian, non-institutionalized population [12]. Participation rates for the 1998 and 2001 NHANES were 89.8% and 77.3%, respectively. At a baseline visit, information on education level was obtained from an adult household member. For each family member, the highest education completed was reported. This data contained unique 13-digit personal identification numbers that were individually linked to mortality data from the Korean National Statistical Office. By law, all Korean deaths must be reported to the Korea National Statistical Office. We identified deaths that occurred in the years 1999–2005 for those in the 1998 NHANES and 2002–2005 for those in the 2001 NHANES. Duration of mortality follow-up was 7.1 years. Similar to that in NHANES, levels of education reported on death certificates were determined by the highest level of education completed. The information on education was based on reports from the decedent's kin.

Covariates considered in our analyses were gender, age, duration between survey and death, and cause of death. Duration between health survey and death was grouped into categories of 3.4 years or less and 3.5–7.1 years to allocate samples equally into groups. Causes of death were coded according to the 10^th ^revision of the *International Classification of Disease *(ICD-10) and grouped as cancer (C00–C97), cardiovascular disease (I00–I99), external causes (V01–Y98), and other.

For our analysis, we first compared education level between NHANES data and death certificate data and calculated the proportion of concordant and discordant pairs. Then we determined sensitivity and specificity of education in death certificate using the standard method, [13] with education reported in NHANES considered the gold standard. Sensitivity and specificity by cause of death were not presented due to the small size of each education group. We also estimated the agreement rates of education level between NHANES data and death certificate data. Overstatement and understatement rates were calculated. Overstatement refers to the inflated education level recorded in death certificates compared with that in NHANES, while understatement means the reverse. Confidence intervals (CI) for sensitivity, specificity, agreement rates, overstatement rates and understatement rates were calculated by asymptotic method. For these analyses, education data from death certificates and NHANES were categorized into five levels (college or higher, high school, middle school, elementary school and no education) and three levels (college or higher, high and middle school, elementary school or less). This was consistent with categorizations used in prior two studies [6,7]. Finally, after adjusting for all covariates, we estimated odds ratios with 95% CI for agreement in educational attainment when five and three educational groups were used. All statistical analyses were performed with SAS statistical software.

## Results

Unique personal identification numbers of 12,801 subjects aged 20+ (6,900 for the 1998 NHANES and 5,901 for the 2001 NHANES) were linked to mortality information. A total of 513 deaths were identified: 376 deaths for the 1998 NHANES and 137 deaths for the 2001 NHANES. Of these, 511 (99.6%) had education levels available from both the death certificate and NHANES. One decedent had no education information in the NHANES and another decedent's education was not recorded in the death certificate. Of the 511 participants included in our analyses, 57.9% (n = 296) were men and 61.1% (n = 312) were aged 65+. The mean age of subjects was 66.4 years (SD 14.4). Decedent age varied with education from 52.7 years (SD 16.3) for those with college education or higher to 75.8 years (SD 9.2) for those with no education. A marked gender difference in education was also found with 24.7% (n = 73) of men and 67.9% (n = 146) having no education recorded in the NHANES. While the proportion of those having college or higher education only reached 2.3% (n = 5) for women, the proportion for men was 8.8% (n = 26).

Table 1 shows the cross-classification of education level by death certificate and the adult household member's report at baseline visit in the NHANES of South Korea. For 511 deaths, 150 (29.4%) discordant pairs were found. Inconsistencies were most common when education was reported as middle school in NHANES. Of 54 participants whose education was reported as middle school in NHANES, only 25 (46.3%) were recorded as having middle school education in death certificate data.

**Table 1:** Comparison of education level between Korea National Health and Nutrition Examination Survey (NHANES) data and death certificate data among 513 decedents

Education recorded on death certificate	Education reported in the 1998 and 2001 NHANES						
	
	College or higher	High school	Middle school	Elementary school	No education	Unknown	Total
College or higher	26 (83.9)	3 (4.9)	1 (1.9)	0 (0.0)	0 (0.0)	0 (0.0)	30
High school	3 (9.7)	42 (68.9)	13 (24.1)	3 (2.0)	3 (1.4)	0 (0.0)	64
Middle school	1 (3.2)	8 (13.1)	25 (46.3)	18 (12.2)	2 (0.9)	0 (0.0)	54
Elementary school	1 (3.2)	7 (11.5)	12 (22.2)	98 (66.7)	44 (20.1)	0 (0.0)	162
No education	0 (0.0)	0 (0.0)	3 (5.6)	28 (19.1)	170 (77.6)	1 (100.0)	202
Unknown	0 (0.0)	1 (1.6)	0 (0.0)	0 (0.0)	0 (0.0)	0 (0.0)	1

Total	31 (100.0)	61 (100.0)	54 (100.0)	147 (100.0)	219 (100.0)	1 (100.0)	513

Note: The denominator for the calculation is the number of deaths classified by level of education noted on the NHANES survey, so that the column percentages add to 100%.

Table 2 presents the sensitivity and specificity of education in death certificate when education level derived from the health survey was considered as the gold standard. The sensitivity and specificity of the death certificate in classifying a decedent as having college or higher education were 0.84 (95% CI: 0.71–0.97) and 0.99 (95% CI: 0.98–1.00), respectively. However, for middle school education, the sensitivity was poor. While the level of sensitivity for high and middle school was greater in men than women, this pattern could not be generalized to other educational groups. A very low sensitivity (0.20) for middle school in women was based on the small sample number (n = 5). With the exception of high school, the sensitivity of education for ages 65+ was similar to that for ages 20–64. In addition, the magnitude of sensitivity and specificity did not vary with the length of time between acquisition of health survey and death certificate data.

**Table 2:** Sensitivity, specificity and their 95% confidence intervals (CI)* of education level recorded in death certificates using education reported in National Health and Nutrition Examination Survey (NHANES) of South Korea as the gold standard (N = 511)

Education	N	Sensitivity (95% CI)	N	Specificity (95% CI)	N	Sensitivity (95% CI)	N	Specificity (95% CI)
	All subjects							
					
College or higher	31	0.84 (0.71–0.97)	480	0.99 (0.98–1.00)				
High and middle school	114	0.77 (0.69–0.85)	397	0.92 (0.90–0.95)				
High school	60	0.70 (0.58–0.82)	451	0.95 (0.93–0.97)				
Middle school	54	0.46 (0.33–0.60)	457	0.94 (0.91–0.96)				
Elementary school or less	366	0.93 (0.90–0.96)	145	0.84 (0.78–0.90)				
Elementary school	147	0.67 (0.59–0.74)	364	0.82 (0.79–0.86)				
No education	219	0.78 (0.72–0.83)	292	0.89 (0.86–0.93)				
	Men				Women			
		
College or higher	26	0.81 (0.66–0.96)	270	0.99 (0.97–1.00)	5	1.00 (1.00–1.00)	210	1.00 (1.00–1.00)
High and middle school	100	0.80 (0.72–0.88)	196	0.90 (0.86–0.94)	14	0.57 (0.31–0.83)	201	0.95 (0.92–0.98)
High school	51	0.71 (0.58–0.83)	245	0.93 (0.89–0.96)	9	0.67 (0.36–0.97)	206	0.98 (0.96–1.00)
Middle school	49	0.49 (0.35–0.63)	247	0.91 (0.88–0.95)	5	0.20 (0.00–0.55)	210	0.97 (0.94–0.99)
Elementary school or less	170	0.91 (0.86–0.95)	126	0.87 (0.81–0.92)	196	0.95 (0.92–0.98)	19	0.68 (0.48–0.89)
Elementary school	97	0.69 (0.60–0.78)	199	0.80 (0.75–0.86)	50	0.62 (0.49–0.75)	165	0.85 (0.79–0.90)
No education	73	0.64 (0.53–0.75)	223	0.92 (0.88–0.96)	146	0.84 (0.78–0.90)	69	0.81 (0.72–0.90)
	Ages 20–64				Ages 65			
		
College or higher	23	0.83 (0.67–0.98)	176	0.99 (0.97–1.00)	8	0.88 (0.65–1.00)	304	0.99 (0.98–1.00)
High and middle school	82	0.77 (0.68–0.86)	117	0.85 (0.78–0.91)	32	0.78 (0.64–0.92)	280	0.96 (0.93–0.98)
High school	44	0.80 (0.68–0.91)	155	0.90 (0.86–0.95)	16	0.44 (0.19–0.68)	296	0.98 (0.96–0.99)
Middle school	38	0.45 (0.29–0.61)	161	0.91 (0.87–0.96)	16	0.50 (0.26–0.75)	296	0.95 (0.92–0.97)
Elementary school or less	94	0.84 (0.77–0.91)	105	0.83 (0.76–0.90)	272	0.96 (0.94–0.98)	40	0.88 (0.77–0.98)
Elementary school	67	0.69 (0.58–0.80)	132	0.80 (0.73–0.86)	80	0.65 (0.55–0.75)	232	0.84 (0.79–0.89)
No education	27	0.52 (0.33–0.71)	172	0.94 (0.91–0.98)	192	0.81 (0.76–0.87)	120	0.83 (0.76–0.89)
	Duration between health survey and death, 3.4 years or less				Duration between health survey and death, 3.5–7.1 years			
		
College or higher	6	1.00 (1.00–1.00)	249	1.00 (0.99–1.00)	25	0.80 (0.64–0.96)	231	0.99 (0.97–1.00)
High and middle school	60	0.80 (0.70–0.90)	195	0.92 (0.89–0.96)	54	0.74 (0.62–0.86)	202	0.93 (0.89–0.96)
High school	33	0.67 (0.51–0.83)	222	0.95 (0.92–0.98)	27	0.74 (0.58–0.91)	229	0.95 (0.92–0.98)
Middle school	27	0.44 (0.26–0.63)	228	0.92 (0.89–0.96)	27	0.48 (0.29–0.67)	229	0.95 (0.92–0.98)
Elementary school or less	189	0.92 (0.88–0.96)	66	0.83 (0.74–0.92)	177	0.94 (0.90–0.97)	79	0.85 (0.77–0.93)
Elementary school	73	0.66 (0.55–0.77)	182	0.81 (0.75–0.86)	74	0.68 (0.57–0.78)	182	0.84 (0.79–0.89)
No education	116	0.77 (0.69–0.84)	139	0.91 (0.86–0.95)	103	0.79 (0.71–0.87)	153	0.88 (0.83–0.93)

*Confidence intervals for sensitivity and specificity were calculated by asymptotic method.

Table 3 shows the overall agreement rate, overstatement rate and understatement rates with 95% CI. When education level was categorized into five groups (college or higher, high school, middle school, elementary school, and no education), the overall agreement rate was 70.7% (95% CI: 66.8%–74.6%). The magnitude of agreement rate for education did not vary with gender, age, and duration between health survey and death. This was generally true for cause specific analyses. The overstatement rate tended to be greater than the understatement rate. The education level of 87 (17.0%) subjects was reported as greater in death certificate than NHANES data while 63 (12.3%) subjects were recorded with lower education in death certificate data than in NHANES data. This was true for both gender, age and causes of death except for external causes. Meanwhile, the understatement rate for external causes tended to be greater than the rates for other three broad causes. When educational level was grouped into five categories, understatement rate for external causes was 20% while understatement rates for other broad causes were about 10%. Based on logistic analysis when the educational level was grouped into three categories, odds ratio of understatement for external causes (reference group = other broad causes) was 2.85 (1.15–7.05).

**Table 3:** Agreement rate (%), overstatement* rate (%), understatement* rate (%), and their 95% confidence intervals (CI)* for education level by gender, age, and cause of death: Reliability between Korea National Health and Nutrition Survey (NHANES) data and death certificate data

	No. of subjects	Agreement rate (95% CI)	Overstatement rate (95% CI)	Understatement rate (95% CI)
**Five educational groups (college or higher, high school, middle school, elementary school, no education)**				
All subjects	511	70.7 (66.8–74.6)	17.0 (13.7–20.3)	12.3 (9.5–15.1)
Gender				
Men	296	65.9 (60.5–71.3)	19.3 (14.8–23.8)	14.9 (10.8–19.0)
Women	215	77.2 (71.6–82.8)	14.0 (9.4–18.6)	8.8 (5.0–12.6)
Age groups				
20–64	199	65.8 (59.2–72.4)	17.6 (12.3–22.9)	16.6 (11.4–21.8)
65+	312	73.7 (68.8–78.6)	16.7 (12.6–20.8)	9.6 (6.3–12.9)
Duration between health survey and death				
3.4 years or less	255	69.4 (63.8–75.1)	19.2 (14.4–24.1)	11.4 (7.5–15.3)
3.5–7.1 years	256	71.9 (66.4–77.4)	14.8 (10.5–19.2)	13.3 (9.1–17.4)
Cause of death				
Cancer	124	71.0 (63.0–79.0)	17.7 (11.0–24.4)	11.3 (5.7–16.9)
Cardiovascular disease	132	69.7 (61.9–77.5)	18.2 (11.6–24.8)	12.1 (6.5–17.7)
External causes	60	65.0 (52.9–77.1)	15.0 (6.0–24.0)	20.0 (9.9–30.1)
Other causes	195	72.8 (66.6–79.0)	16.4 (11.2–21.6)	10.8 (6.4–15.2)
**Three educational groups (college or higher, high and middle school, elementary school or less)**				
All subjects	511	88.9 (86.2–91.6)	5.9 (3.9–7.9)	5.3 (3.4–7.2)
Gender				
Men	296	86.2 (82.3–90.1)	6.8 (3.9–9.7)	7.1 (4.2–10.0)
Women	215	92.6 (89.1–96.1)	4.7 (1.9–7.5)	2.8 (0.6–5.0)
Age groups				
20–64	199	80.9 (75.4–86.4)	8.5 (4.6–12.4)	10.6 (6.3–14.9)
65+	312	93.9 (91.2–96.6)	4.2 (2.0–6.4)	1.9 (0.4–3.4)
Duration between health survey and death				
3.4 years or less	255	89.4 (85.6–93.2)	6.3 (3.3–9.2)	4.3 (1.8–6.8)
3.5–7.1 years	256	88.3 (84.3–92.2)	5.5 (2.7–8.3)	6.3 (3.3–9.2)
Cause of death				
Cancer	124	84.7 (78.4–91.0)	11.3 (5.7–16.9)	4.0 (0.6–7.4)
Cardiovascular disease	132	87.1 (81.4–92.8)	6.8 (2.5–11.1)	6.1 (2.0–10.2)
External causes	60	86.7 (78.1–95.3)	1.7 (0.0–5.0)	11.7 (3.6–19.8)
Other causes	195	93.3 (89.8–96.8)	3.1 (0.7–5.5)	3.6 (1.0–6.2)

*Overstatement refers to the inflated educational level recorded in death certificate compared with that in NHANES, while understatement means the reverse. Confidence intervals for agreement rate, overstatement rate, and understatement rate were calculated by asymptotic method.

As presented in Table 3, the magnitude of reliability increased as the number of educational groups decreased. When the education level was categorized into three groups (college or higher, high and middle school, elementary school or less), the overall agreement rates was 88.9% (95% CI: 86.2%–91.6%). For this categorization of education, overstatement and understatement rates diminished and there was no marked evidence of education inflation in death certificate data compared with health survey.

Table 4 presents odds ratio (95% CI) of agreement when five and three educational groups were used to assess agreement. When five educational groups were used, the middle and elementary school education groups showed a significantly smaller likelihood of agreement after adjusting for gender, age (10-year age), duration between health survey and death, and cause of death. However, this was not true when assessing agreement with three educational groups. No significant difference in odds ratio was found for gender, age, duration between health survey and deaths, and cause of death, likely in part due to small sample size.

**Table 4:** Odds ratio and 95% confidence interval (CI) of agreement in education level (N = 511)

	OR (95% CI) of agreement for five educational groups	OR (95% CI) of agreement for three educational groups
Gender		
Men	1.00 (reference)	1.00 (reference)
Women	1.47 (0.93–2.31)	1.27 (0.65–2.50)
Age groups		
10-year age (continuous)	1.10 (0.92–1.31)	1.20 (0.95–1.52)
Duration between health survey and deaths		
3.4 years or less	1.00 (reference)	1.00 (reference)
3.5–7.1 years	1.09 (0.74–1.63)	0.92 (0.52–1.65)
Education in health survey		
College or higher	1.00 (reference)	1.00 (reference)
High school	0.46 (0.15–1.41)	0.58 (0.20–1.73)
Middle school	0.17 (0.06–0.51)	0.58 (0.20–1.73)
Elementary school	0.33 (0.12–0.95)	1.59 (0.51–5.00)
No education	0.47 (0.15–1.45)	1.59 (0.51–5.00)
Cause of death		
Cancer	1.00 (reference)	1.00 (reference)
Circulatory disease	0.81 (0.46–1.42)	1.00 (0.48–2.11)
External causes	0.89 (0.44–1.78)	1.74 (0.68–4.47)
Other causes	0.91 (0.54–1.55)	2.00 (0.92–4.35)

Note: all variables were entered into logistic models simultaneously.

## Discussion

Results of this study showed that the proportion of deaths without recorded education was very low, only one of 513 (0.2%) in death certificate, far less than prior US studies in which information on education for about 15%–27% of all deaths occurred was not available in the death certificate [1,2]. According to death certificate data covering all South Koreans, the rate of failure to report the decedent's educational attainment was 0.3% for men and 0.4% for women aged 35–64 [6]. This may be due to the Korean death certification system where any vague or missing item on the death certificate is further clarified by the Korea National Statistical Office via telephone [6]. This low percentage of missing education information in South Korean death certificate data could provide a great potential to monitor socioeconomic inequalities in mortality.

Direct comparison of the validity and reliability level found in this study with prior reports in other countries may be problematic because of differences in age distribution and educational categories used. Factors associated with participation to health survey may also affect the overall validity and reliability. Nevertheless, we may conclude that this study showed a similar or higher level of validity and reliability in education information than in Western studies. For example, the agreement rate for three educational groups (less than high school graduate, high school graduate, and greater than high school education) in a prior study was 84% [1]. Results of this study showed an agreement rate of 88.9% for the same categories. A prior study presented a sensitivity of 0.85 for college or higher education, [2] which is similar to what was found in this study (0.84). It should be noted that in prior studies, a substantial number of subjects with missing education information (15%–27%) were found but were eliminated from analyses that reported the agreement rate and sensitivity [1,2].

Despite the relatively high level for overall validity and reliability, low sensitivity and agreement rates were found in this study for participants with middle school education. After adjusting for covariates, middle and elementary school education groups were less concordantly recorded in death certificate data. One possible explanation for this may be related to historical changes in the education system in South Korea. During Japanese colonial occupation (1910–1945) and the Korean War (1950–1953), South Korea could not establish a nationwide educational curriculum [14]. An education system divided by elementary school (6 years), middle school (3 years), and high school (3 years) began in 1954 when the Ministry of Education of Korea first introduced an official nationwide curriculum. Various types of educational attainment, which are difficult to group into the categories used in this study, would be inevitable among decedents whose childhood and adolescent education was achieved before the modern South Korean educational system was established. Difficulties in determining one's educational level have also remained in vital statistic records. For example, middle and high school levels were grouped as one category in early 1990s death certificate data [7]. However, when middle school and elementary school education were collapsed into high school and no education respectively, statistically significant differences in agreement of education did not appear.

It was hypothesized that male decedents' education would be more accurately reported by surviving female spouses than female decedents' due to the gender gap in life expectancy [3]. However, this study showed that the agreement rate of education for women was generally greater than that for men. Statistically significant gender differences in odds of agreement were found for both educational categories (five and three groups) when only the gender variable was entered into logistic model (data not shown). In South Korea a woman's death is more frequently reported by her children, who may know more about her education than other informants. The widow's cognitive or communication problems may lower the agreement rate of male decedents' education. However, the comparison of accuracy level between widow's reporting (for male decedents' education) and children's reporting (for female decedents' education) needs to be further explored. If a woman's informants were uncertain as to which education levels she achieved, it might be difficult to accurately deduce and report the actual education level. However, in this study reports of women's education in the NHANES were concentrated around the "no education" level (67.9%). The simplicity of a woman's education might help informants for death certification to report education information accurately. Based on logistic model, significantly greater odds ratios of agreement for women compared with men became insignificant after adjusting for education (data not shown).

Although the magnitude of overall agreement rate for education did not vary with causes of death, understatement rate for external causes tended to be greater than the rates for other broad causes. Higher understatement rate for external causes was mainly due to stigmatized causes of death rather than other external causes such as transport accident. Understatement rate for suicide (n = 17) was 35.3% (n = 6) while the rate for transport accident (n = 24) was 8.3% (n = 2). Logistic analysis presented a greater odds ratio of understatement for suicide (data not shown). This suggests that a considerable understatement for education may occur in stigmatized causes of death.

Overstatement and understatement of education by descendants may vary with regions with different cultural background. Results of this study showed a tendency for inflation of education when grouped into five categories but did not reveal any evidence of inflation when education was collapsed into three categories. These findings agree with prior US studies where inflation was found in six educational groups [3] but did not appear in collapsed educational groups [1]. Thus, when using unlinked data a more collapsed categorization of education would produce a more valid result on estimating mortality inequality. However, as the sensitivity of college or higher education was generally greater than that of no education and the pattern did not vary with covariates, it can be suggested that educational mortality differentials between no education and college or higher education in the previous study [6] were underestimated. Indeed, several longitudinal mortality follow-up studies presented a substantial difference in mortality by education in South Korea [15,16]. Given that the level of agreement did not significantly vary with cause of death, cause-specific analysis may not result in biased results. However, a more collapsed categorization in education would be recommended especially when a more definitive conclusion regarding educational mortality inequality is required.

## Conclusion

The low percentage of missing education information in South Korean death certificate data could provide a great potential to monitor socioeconomic inequalities in mortality. Despite the relatively high level for overall validity and reliability, a more collapsed categorization of education would produce a more valid result on estimating mortality inequality when using unlinked data.

## List of abbreviations

CI, confidence intervals; NHANES, National Health and Nutrition Examination Survey of South Korea; SD, standard deviation.

## Competing interests

The author(s) declare that they have no competing interests.

## Authors' contributions

YHK conceived and designed the study with advice from HRK and JWL. YHK analyzed and interpreted the data and drafted the paper. HRK and JWL interpreted the data and critically revised the draft of the paper. All authors read and approved the final manuscript.

## Pre-publication history

The pre-publication history for this paper can be accessed here:


